# Fungal Diversity in Intertidal Mudflats and Abandoned Solar Salterns as a Source for Biological Resources

**DOI:** 10.3390/md17110601

**Published:** 2019-10-23

**Authors:** Young Mok Heo, Hanbyul Lee, Kyeongwon Kim, Sun Lul Kwon, Min Young Park, Ji Eun Kang, Gyu-Hyeok Kim, Beom Seok Kim, Jae-Jin Kim

**Affiliations:** 1 Division of Environmental Science & Ecological Engineering, College of Life Sciences & Biotechnology, Korea University, Seoul 02841, Korea; hym011@korea.ac.kr (Y.M.H.); hblee95@korea.ac.kr (H.L.); rudndjs@korea.ac.kr (K.K.); sun-lul@korea.ac.kr (S.L.K.); lovewood@korea.ac.kr (G.-H.K.); 2 Department of Biosystems & Biotechnology, College of Life Sciences and Biotechnology, Korea University, Seoul 02841, Korea; min0@korea.ac.kr (M.Y.P.); heyyo9725@korea.ac.kr (J.E.K.); 3 Division of Biotechnology, College of Life Sciences & Biotechnology, Korea University, Seoul 02841, Korea; biskim@korea.ac.kr

**Keywords:** fungal community, marine fungi, phylogenetic analysis, saltwork, tidal flat

## Abstract

Intertidal zones are unique environments that are known to be ecological hot spots. In this study, sediments were collected from mudflats and decommissioned salterns on three islands in the Yellow Sea of South Korea. The diversity analysis targeted both isolates and unculturable fungi via Illumina sequencing, and the natural recovery of the abandoned salterns was assessed. The phylogeny and bioactivities of the fungal isolates were investigated. The community analysis showed that the abandoned saltern in Yongyudo has not recovered to a mudflat, while the other salterns have almost recovered. The results suggested that a period of more than 35 years may be required to return abandoned salterns to mudflats via natural restoration. Gigasporales sp. and *Umbelopsis* sp. were selected as the indicators of mudflats. Among the 53 isolates, 18 appeared to be candidate novel species, and 28 exhibited bioactivity. *Phoma* sp., *Cladosporium sphaerospermum*, *Penicillium* sp. and *Pseudeurotium bakeri*, and *Aspergillus urmiensis* showed antioxidant, tyrosinase inhibition, antifungal, and quorum-sensing inhibition activities, respectively, which has not been reported previously. This study provides reliable fungal diversity information for mudflats and abandoned salterns and shows that they are highly valuable for bioprospecting not only for novel microorganisms but also for novel bioactive compounds.

## 1. Introduction

As interest in marine living resources worldwide has increased, research on marine fungi has progressed considerably over the past two decades, including the discovery of new species and novel natural compounds [1]. It is still insufficient compared to the research on terrestrial fungi, but the study of marine fungi has been extended to intertidal zones such as mangrove forests and coastal wetlands [2,3]. In addition, there are other unique intertidal environments such as mudflats and abandoned salterns.

There are different types of tidal flats, and mudflats are differentiated from sandy tidal flats. Mudflats are a kind of coastal salt marsh made of clay deposited by waves and rivers and are a unique environment as they are exposed to the atmosphere twice a day depending on the tide. In the Yellow Sea of South Korea, mudflats are distributed widely along the coastline. The Ministry of Oceans and Fisheries (MOF, South Korean government) reported that the total area of mudflats in 2013 was 2487.2 km^2^, which is approximately 2.5% of the national territory [4]. Although the area has decreased by 22.4% since 1987 due to the mudflat reclamation project for the expansion of agriculture land, recent efforts have been made to preserve and restore mudflat areas. For example, the MOF expanded the protected mudflat area from 79.62 km^2^ to approximately 1265.46 km^2^ in 2018 [5]. The coast of the Yellow Sea has topographical conditions that are suitable for solar salt production, as the coastal slope is very low. A large number of solar salterns have been constructed and operated on the coast throughout history. A broad area of the intertidal mudflat had even been reclaimed for this purpose. However, in the 1980s, people were informed that mudflats have great ecological and economic value, so citizens and environmental scientists insisted on restoring the mudflats. Mudflats are an ecological hot spot that performs numerous ecological functions, such as providing habitat for shellfish, water birds, and migratory birds. According to the Korea Marine Environment Management Corporation (KOEM, South Korean government), the economic value of the Korean mudflats was estimated at approximately 15 billion dollars per year in 2017, including value from food production, waste treatment, recreation, habitat/refugia, disturbance regulation, and conservation [6]. In addition, people were concerned about consuming solar salt, as global concerns about marine pollution and its negative effects on human health were raised. In this context, most of the salterns were closed because of the government’s policy as well as the decrease in market demand, and the embankments of the closed salterns were withdrawn to allow the natural recovery of the mudflats [5]. As a result, these abandoned salterns have become a unique environment where the tides have returned to the salterns installed in the dry and windy areas of the intertidal zone.

Because of strong selective pressure, environments that are unique or disadvantageous for survival are likely to have novel microorganisms as well as novel secondary metabolites [7,8]. Therefore, microorganisms living in these unusual environments, especially fungi, which have been considered a treasure trove of bioactive secondary metabolites, are highly valuable to investigate. Many studies have been reported on saltern extremophiles and microorganisms in the mangrove forest, another intertidal environment [9,10,11,12]. Until now, however, studies of the diversity, physiology, and bioactivity of fungi in intertidal mudflats and abandoned salterns have rarely been reported in the overall field. To the best of our knowledge, there are only two studies on the fungal diversity of these environments: one on the diversity of *Aspergillus* in a mudflat and one on the diversity of mycorrhizal fungi in an abandoned saltern [13,14]. Therefore, it is necessary to investigate the microbial communities of these environments. Through community analysis, a recovery assessment of the decommissioned salterns is also possible; there has been no study on the recovery of the microbial community in this environment to date [15]. Even in other similar sites around the world, few studies on macrofauna/flora have been reported, and there has been no study of the microorganisms, which are the cornerstone of the ecosystem. In fact, it has been proven by many researchers in recent decades that microbial community analysis is a useful tool for ecosystem assessment. Most such studies have used bacterial communities because of the large bacterial DNA database that is available. However, a large amount of fungal DNA data has now been stored as well. It is also necessary to evaluate these environments as biological resources. Studies on the fungi isolated from mudflats and their bioactive secondary metabolites are also rare. To date, *Chaetomium cristatum*, *Fusarium oxysporum*, *Aspergillus niger*, *Thielavia hyalocarpa*, *Thielavia* sp., and *Paecilomyces formosus* have been reported and are known to produce cristazine (cytotoxic and antioxidant), oxysporizoline (antibacterial), 6,9-dibromoflavasperone (antioxidant), 1-*O*-(α-d-mannopyranosyl)geraniol (a biotransformation product of geraniol), thielaviazoline (antibacterial and antioxidant), and formoxazine (antibacterial and antioxidant), respectively [16,17,18,19,20,21]. The lack of studies is likely primarily due to the limited global distribution of mudflats and salterns. Another possible reason is that it is difficult to isolate a large number of fungi due to the extreme environmental conditions; the environment dramatically changes between aerobic and anaerobic conditions twice a day, and dry stress occurs at the ebb of the tide due to the weather conditions described above.

The aims of this study were 1) to analyze the fungal community and diversity in the intertidal mudflats and the abandoned solar salterns of the Yellow Sea in South Korea; 2) to assess the natural recovery of the abandoned salterns to mudflats and determine the indicator taxa; and 3) to evaluate these environments for the bioprospecting of novel species and bioactive compounds.

## 2. Results and Discussion

### 2.1. The Fungal Community in Intertidal Mudflats and Abandoned Salterns

**Figure 1:**
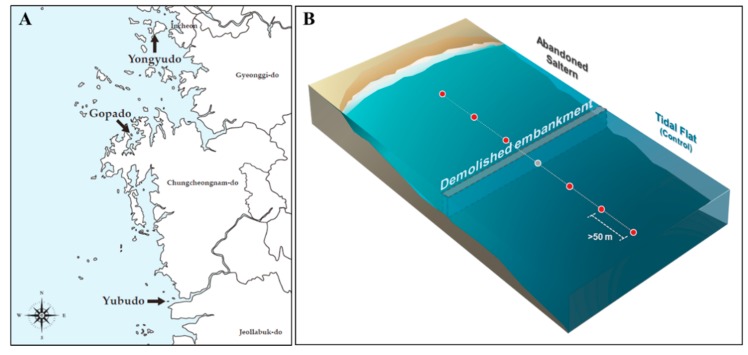
(**A**) Locations of the three sampling sites and an illustration of abandoned salterns and mudflats in the west coast of South Korea. (**B**) Illustration of abandoned salterns and tidal flats in the west coast of South Korea. The circles indicate the sampling points.

Intertidal sediments were sampled from mudflats and abandoned salterns in Yongyudo, Gopado, and Yubudo located on the coast of the Yellow Sea in South Korea (Figure 1).

#### 2.1.1. Fungal Diversity and Recovery Assessment

The fungal communities and diversity in these intertidal environments were investigated by high-throughput sequencing of the sediment samples. Through the community analysis, the natural recovery of the decommissioned salterns was also assessed.

**Figure 2:**
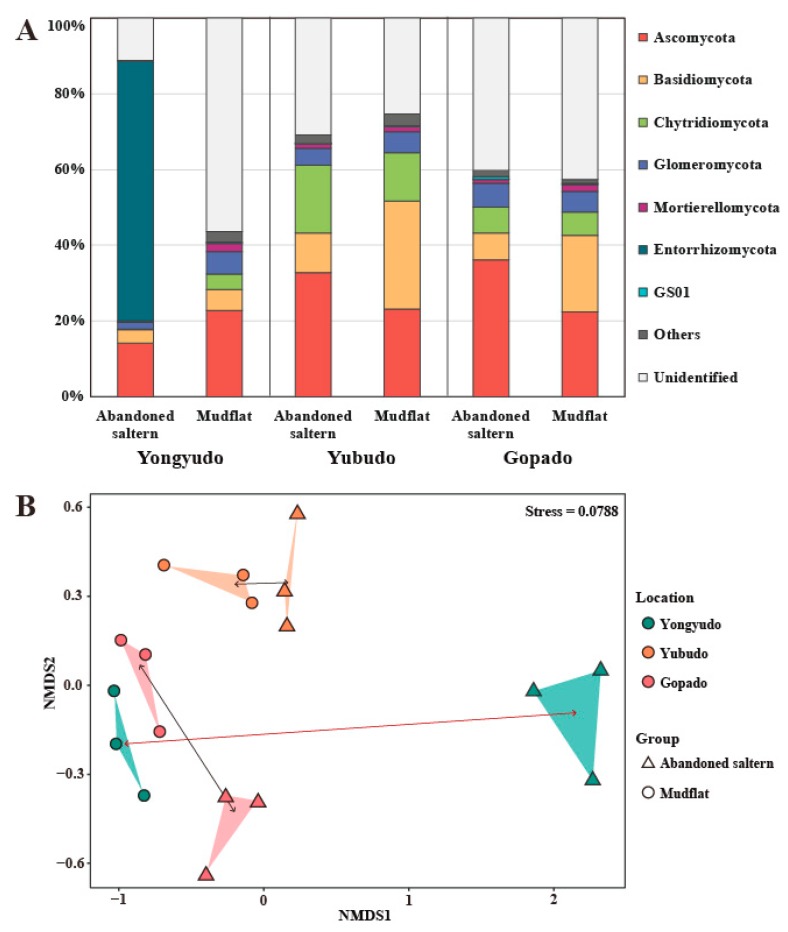
(**A**) Fungal community composition of intertidal mudflats and abandoned salterns in Yongyudo, Yubudo, and Gopado at the phylum level. Mean values of the abundance are used. (**B**) Nonmetric multidimensional scaling plot of the fungal community in intertidal mudflats and abandoned salterns in Yongyudo, Yubudo, and Gopado.

At the phylum level of the fungal community composition, Ascomycota and Basidiomycota, the two major phyla of the kingdom Fungi, were the dominant phyla (Figure 2A). Meanwhile, the community composition of the abandoned saltern in Yongyudo was clearly different. It was highly dominated by Entorrhizomycota, while Chytridiomycota and Mortierellomycota dominated the other abandoned salterns and mudflats. Considering that the phylum Entorrhizomycota is composed of plant pathogenic fungi, the difference may be due to the introduction of their host plants at the initial stage of ecological succession after the saltern was abandoned. Meanwhile, a large number of fungi could not be identified, indicating a lack of DNA-based phylogenetic information on fungi in these intertidal environments. As the community compositions of the other salterns and mudflats were similar to each other except for that of the saltern in Yongyudo, a nonmetric multidimensional scaling (NMDS) plot was constructed using the database of fungal operational taxonomical units (OTUs) and their abundance to determine the distance between the fungal communities. According to the NMDS results, it appeared that the distance between the abandoned saltern and the mudflats in Yongyudo was much longer than the distance between the two environments on the other islands (Figure 2B). This indicates that the abandoned salterns in Yongyudo that were abandoned less than a year ago were the least recovered to mudflat. Since the salterns of Yubudo and Gopado were abandoned 20 and 35 years ago, respectively, it was expected that natural recovery would have occurred. This was supported by the short distance between the communities of the two salterns and those of nearby mudflats on the NMDS plot. Thus, we clustered the samples by the expected recovery status, consisting of recovered saltern (RS), nonrecovered saltern (NS), and mudflat. In fact, the NMDS plot showed that the fungal communities of the two environment types in Yubudo and Gopado were almost similar but were not completely clustered, especially in Gopado. This indicated that the fungal communities of the abandoned salterns were not fully recovered even after 35 years. Similarly, Bernhard and his colleague reported that salt marshes that were impounded and subsequently restored took more than 30 years to recover and become similar to the neighboring tidal flats [22]. They suggested that the regular inflow of tidal water increases the stability of the ecosystem. The abandoned salterns require a great deal of time to return to their original state due to the disturbance of human activity blocking the tidal water for a long time, resulting in a significant decrease in stability of microbial communities. Thus, with natural restoration alone, an artificially isolated environment requires an extremely long time to construct a microbial ecosystem similar to that of the adjacent environment, and it may require more than 35 years in the case of the abandoned salterns.

To verify the differences between the fungal communities, Shannon-Wiener and Gini-Simpson indices of each community were calculated. These two indices have been commonly used to calculate α-diversity, and the value of Shannon-Wiener and Gini-Simpson indices are more affected by species richness and evenness, respectively. The α-diversity indices of the NS were significantly lower in both the indices compared to those of the other environments, as expected (Table 1). This indicates that artificially blocking tidal inflow and conducting solar salt production reduce the species richness and evenness of the microbial community in the saltern, and the reduced diversity does not recover in a short period of time.

We tested the significance of the difference by the clustered group using the Kruskal-Wallis rank sum test. There was no significant difference between the α-diversity indices of RSs and mudflats, while those of the NS were significantly lower than those of the others. The abandoned saltern in Yongyudo was an environment continuously interrupted by humans until recently. Thus, the ecosystem of the NS could be easily dominated by a small number of species that could survive or adapt under this harsh condition, resulting in significantly lower microbial diversity. In fact, the diversity indices of RSs and mudflats were high when compared to those of fungal communities in other environments, such as livestock manure, compost, and soil [23,24,25,26]. This implies that diverse fungi exist in these unique environments, thus making them valuable for bioprospecting.

#### 2.1.2. Determination of Indicator Taxa

We tried to determine the major differences among the fungal communities. Various analytical methods were used to find indicator taxa that are responsible for the differences, including linear discriminant analysis effect size (LEfSe), indicator species analysis (ISA), RandomForest, and mvabund.

**Figure 3:**
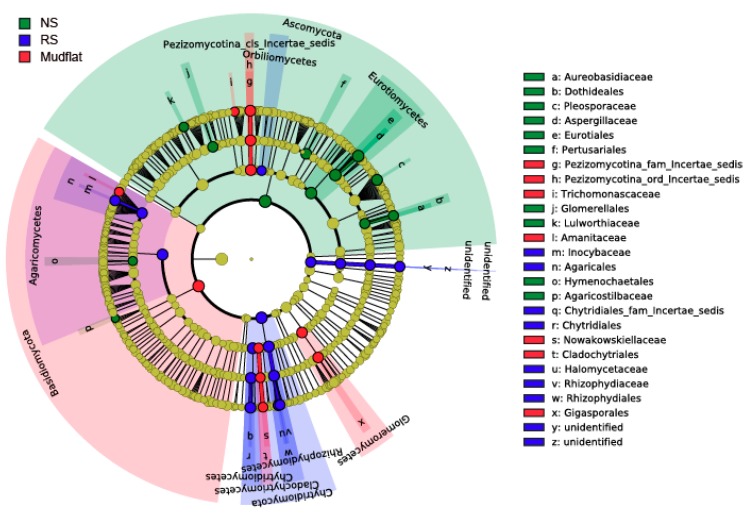
Linear discriminant analysis effect size (LEfSe) cladogram of fungal composition in abandoned salterns and mudflats (logarithmic linear discriminant analysis (LDA) score > 2, *p* < 0.05). Red, green, and blue nodes/shades indicate taxa that are significantly higher in relative abundance. The diameter of each node is proportional to the taxon’s abundance. NS and RS mean non-recovered saltern and recovered saltern, respectively.

First, we visualized the result of the Kruskal-Wallis rank sum test with a LEfSe cladogram (Figure 3 and Figure S1). It was obvious that the abundance of Ascomycota differs significantly between NS and the other environmental groups, tending to be lower in NS (Figure S2). RS and mudflat both tended to be abundant in Chytridiomycota and Basidiomycota, but many of the taxa overlapped each other. This finding supports that there is no significant difference between RSs and mudflat, and the RSs were in the process of recovery to mudflats.

For the next step, we investigated the taxa specific to each group via ISA. ISA results in an “IndVal score” calculated by multiplying the relative abundance by the relative frequency of a taxon in each group. Microbial community data are known to be zero-rich and to have many rare species. In this case, OTUs with low mean abundance tend to have low mean variance; this can lead to statistical misinterpretations if some rare species are highly specific to a certain environment. Therefore, the results with low frequency have low fidelity, and taxa with frequencies less than 5 were screened out. As a result, several lineages of taxa were selected to be specific to each environment (Table 2).

**Figure 4:**
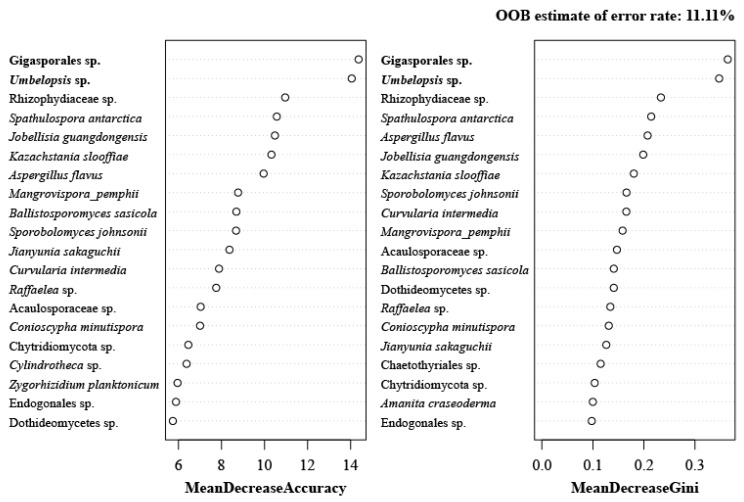
RandomForest importance plot analyzed using fungal abundance data at the species level.

RandomForest analysis was carried out to determine whether there is a hierarchy-based relationship in the fungal community that can distinguish the sample groups. The results are shown as the mean decrease in accuracy and Gini impurity, which indicates the importance of the taxa in the generated decision tree. At the class to species level, two lineages of taxa, Gigasporales sp. and *Umbelopsis* sp., were repeatedly ranked at the top (Figure 4).

Finally, mvabund was used to determine which taxon abundances differed significantly by group. Recently, it was proved that model-based analysis of multivariate abundance data combined with negative binomial regression is excellent for detecting multivariate effects that otherwise would lead to statistical misinterpretation and is powerful when selecting indicator species using multivariate abundance data [27]. Several lineages of taxa, including the order Gigasporales and the family Umbelopsidaceae, appeared to differ significantly among the groups (Table 3).

Various analyses were conducted to determine indicator taxa, but the results of these analyses were not identical. Therefore, the indicator species supported by multiple analyses were selected. Gigasporales sp. was supported by every analysis except for ISA, and *Umbelopsis* sp. was selected in every analysis except for LEfSe. Thus, these two fungal lineages can be regarded as definite indicator taxa for mudflats. In fact, the order Gigasporales belongs to the Glomeromycetes (Glomeromycota), the arbuscular mycorrhizal fungi. It was speculated that the abundance of Gigasporales sp. increases as it forms a symbiotic network with the indigenous halophytes of mudflats and as the abandoned saltern is restored to a mudflat [28]. Meanwhile, Entorrhizomycetes was selected by both ISA and mvabund as well as by a simple visual assessment of the fungal community composition (Figure 2A). However, it was difficult to determine the indicator taxa for the NS because only one sampling site, the abandoned saltern in Yongyudo, represented the NS environment. In other words, this plant pathogenic fungus may be dominant there because of the existence of its unique host plants. Therefore, it can be regarded as an indicator taxon of the NS in Yongyudo but not of all the NSs in the Yellow Sea.

#### 2.1.3. Diversity of Culturable Fungi

The community analysis implied that these intertidal environments are valuable for bioprospecting. Thus, the culturable fungi were isolated from the sediments to evaluate their diversity and to compare the results of metagenome analysis.

A total of 53 fungal strains was isolated from the sediments of the intertidal mudflats and the abandoned salterns (Table 4). It was obvious that the number of fungal isolates was much higher in Yongyudo than in the other two regions. It was suspected that this could be due to the sediment properties, such as the texture and grain size, since there was no significant difference in the environmental factors among sampling locations (Figure S1). The genera *Talaromyces* and *Trichoderma* were mostly isolated from the mudflat samples, and most *Penicillium* spp. were found in the NS. Meanwhile, the diversity and the number of fungal isolates in the NS were much higher than those of the other salterns, which was completely opposite to the result of the metagenome analysis, where the diversity of the NS was the lowest (Table 1, Table 4 and Table S1). In addition, all of the isolates except for *Phanerochaete chrysosporium* KUC10791 belong to Ascomycota. These results support that the actual diversity of environmental microorganisms must be investigated by eDNA-based metabarcoding approaches, unless all possible culture conditions and isolation methods are employed [29].

**Figure 5:**
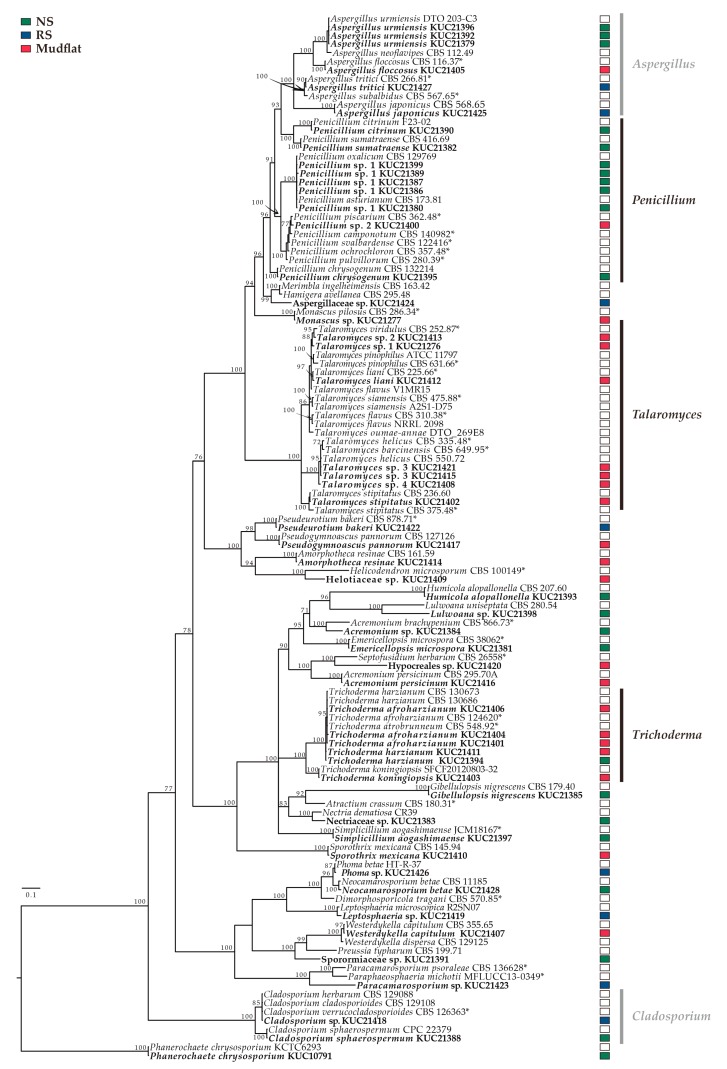
The Bayesian analysis tree based on ITS. Bayesian posterior probabilities (PP) at the nodes are presented if > 75. Type strains are indicated as *. The fungi isolated from this study are in bold. The scale bar indicates the number of nucleotide substitutions per position. NS and RS mean non-recovered saltern and recovered saltern, respectively.

All 53 strains were grouped into 43 groups by morphological analysis and ITS sequences. The best-fit model of ITS sequences is a general time reversible (GTR) + proportion of invariable sites (I) + gamma distribution (G) by MrModeltest and contains 121 taxa and 844 nucleotide characters. In the phylogenetic analysis, 53 strains were classified into 2 phyla, 5 classes, 11 orders, 20 families, 22 genera, and 43 species, based on current taxonomic concepts. The dominant genera of the ITS tree were *Penicillium (*number of strains *=* 9), followed by *Talaromyces* (number of strains = 7), *Aspergillus* (number of strains = 6), *Trichoderma* (number of strains = 6), and *Cladosporium* (number of strains = 2) (Figure 5). *Talaromyces* and *Trichoderma* were the most complex clades and had low resolution. Thus, to precisely perform phylogenetic analysis of *Trichoderma* and *Talaromyces*, EF1-α for *Trichoderma* species and *benA* for *Talaromyces* species were amplified, and phylogenetic trees were constructed (Figure S3 and S4). The best-fit model of EF1-α for *Trichoderma* is Hasegawa-Kishino-Yano (HKY) + I + G and contains 41 taxa and 794 nucleotide characters. Multiple sequence alignments of two loci (ITS & *benA*) were analyzed for *Talaromyces*. Both loci were assigned GTR + I + G as the best fit model, and concatenated datasets contained 64 taxa and 1,155 nucleotide characters (ITS, nchar = 598; *benA*, nchar = 557). Through the EF1-α phylogenetic analysis of *Trichoderma*, KUC21406, KUC21404, KUC21401, KUC21411, and KUC21394 are confidently classified as *T. afroharzianum* and *T. harzianum* in the EF1-α tree. *Talaromyces* sp. 2 KUC21413 was closely related to *T. viridulus* in the ITS tree. In the concatenated tree (ITS & *benA*), however, the position changed to be near *T. galapagensis* with low posterior probability. Therefore, *Talaromyces* sp. 2 KUC21413 was assigned as a novel species candidate. *Talaromyces* sp. 1 KUC21276 appeared to be closely related to *T. angelicus* (99.81% sequence similarity in ITS; 93.22% in benA). *Talaromyces* sp. 3 KUC21415 and *Talaromyces* sp. 3 KUC21421 appeared to be closely related to *T. helices* (99.81% sequence similarity in ITS; 96.7% in *benA*). *Talaromyces* sp. 4 KUC21408 appeared to be closely related to *T. boninensis* (97.97% sequence similarity in ITS; 93.72% in *benA*). Thus, they are suggested to be candidate novel species.

The dominant taxa of the intertidal sediments were *Penicillium* species (number of strains = 8) and *Talaromyces* species (number of strains = 6). A number of fungal isolates belonged to Eurotiomycetes, followed by Sordariomycetes. A total of 18 candidate novel fungal species was isolated from this study. This result demonstrates that many novel fungal candidates remain unexploited in abandoned salterns or intertidal mudflat sediments.

### 2.2. Biological Activities of Fungi from Intertidal Mudflats and Abandoned Salterns

To evaluate the potential value of the intertidal environments for bioprospecting, a variety of biological activities of the fungal extracts were investigated.

#### 2.2.1. Antioxidant Activity

It is well known that reactive oxygen species (ROS) and excessive free radicals cause oxidative chain reactions that damage biomolecules such as proteins and DNA and are responsible for a wide variety of diseases and aging [30,31]. Many antioxidants scavenging free radicals have been discovered, and fungi are one of the major sources of these compounds. However, only four antioxidants were discovered from mudflat-derived fungi: cristazine, 6,9-dibromoflavasperone, thielaviazoline, and formoxazine from *Chaetomium cristatum*, *Aspergillus niger*, *Thielavia* sp., and *Paecilomyces formosus*, respectively [16,18,20,21]. In this study, the extracts of 68 fungi isolated from intertidal mudflats and abandoned salterns were screened for their antioxidant capacity. Among the fungal extracts, three *Aspergillus* spp. (*A. floccosus* KUC21405, *A. japonicas* KUC21425, and *A. urmiensis* KUC21396), four *Penicillium* spp. (*P. chrysogenum* KUC21395 and *Penicillium* sp. 1 KUC21386, KUC21387, and KUC21389), *Phoma* sp. KUC21426, and *Talaromyces liani* KUC21421 exhibited high radical-scavenging activity in both assays using 2.2’-azino-bis-3-ethylbenzothiazoline-6-sulfonic acid (ABTS) radicals and 2,2-diphenyl-1-picrylhydrazyl (DPPH) radicals as substrates, respectively (Table 5).

Several antioxidants have been reported from the genus *Aspergillus*, for example, 3,3‘‘-dihydroxyterphenyllin, 3-hydroxyterphenyllin, candidusin B, and dihydroxymethyl pyranone from *A. candidus* [32,33]; 1,8-dihydroxynaphthalene melanin from *A. bridgeri* [34]; methyl 4-(3,4-dihydroxybenzamido)butanoate, 5-O-methylsulochine, methyl 2-(2,6-dimethoxy-4-methylbenzoyl)-3,5-dihydroxybenzoate, methyl-2-(2,6-dihydroxyl-4-methylbenzoyl)-3-hydroxy-5-methoxybenzoate, physcion, 4-(3,4-dihydroxybenzamido)butanoic acid, and (E)-N-(2-hydroxy-2-(4-hydroxyphenyl)ethyl)-3-(3-hydroxy-4-methoxyphenyl)acrylamide from *A. wentii* [35]. However, there is no report of antioxidants from *A. floccosus*, *A. japonicus*, or *A. urmiensis* that showed the strong radical-scavenging activity seen in our results. The genus *Penicillium* has been reported to produce antioxidants, for example, atrovenetin from *P. atrovenetum*, *P. herquei*, and *P. simplicissimum* (previously known as *P. paraherquei*) [36,37,38]; 2,3,4-trimethyl-5,7-dihydroxy-2,3-dihydrobenzofuran and gentisic acid from *P. citrinum* [39]; and 2,3-dihydroxy benzoic acid from *P. roquefortii* [40]. An ethanol extract of *P. chrysogenum* showing moderate radical-scavenging activity in our results showed high activity in a previous study [41]. Because bisvertinolone has been reported in this species and many bisorbicillinoids are known to be antioxidants, the antioxidant of this species is likely to be bisvertinolone [42]. In the case of the genus *Phoma*, there is only one report of antioxidant compounds, for example, bromochlorogentisylquinones A and B, chlorogentisyl alcohol, and gentisyl alcohol from *P. herbarum* [43]. Some antioxidants have been reported from the genus *Talaromyces*, for example, 8-hydroxyconiothyrinone B, 8,11-dihydroxyconiothyrinone B, 4R,8-dihydroxyconiothyrinone B, 4S,8-dihydroxyconiothyrinone B, and 4S,8-dihydroxy-10-O-methyldendryol E from *T. islandicus* [44]; pentalsamonin from *T. purpureogenus* [45]; N-(4-hydroxy-2-methoxyphenyl) acetamide from *T. funiculosus* [46]; and 6-methylbiphenyl-3,3′,4,5′-tetraol and desmethylaltenusin [47].

*A. japonicas* KUC21396, *Penicillium* sp. 1 KUC21386 and KUC21389, and *Phoma* sp. KUC21426 extracts exhibited even higher ABTS radical-scavenging activity than the positive control, ascorbic acid. In particular, the IC_50_ values of both ABTS and DPPH radical-scavenging activity of *Phoma* sp. KUC21426 extract were significantly lower than the values of other extracts, implying its high antioxidative capacity. To the best of our knowledge, this is the second report of radical-scavenging activity of the genus *Phoma*, which contains a number of plant pathogen species.

#### 2.2.2. Tyrosinase Inhibitory Activity

A total of five fungal extracts (*Cladosporium sphaerospermum* KUC21388, *Lulwoana* sp. KUC21398, *P. citrinum* KUC21390, *Trichoderma afroharzianum* KUC21411, and *Westerdykella capitulum* KUC21407) showed tyrosinase inhibitory activity (Table 5). Tyrosinase is able to oxidize l-3,4-dihydroxyphenylalanine (l-DOPA) to dopaquinone, which is eventually converted to pheomelanin [48]. As melanin biosynthesis is responsible for darkening of the skin tone, researchers have tried to discover tyrosinase inhibitors. Tyrosinase inhibitors can suppress melanin formation in the skin, so they can be developed as whitening agents. In particular, kojic acid, the most widely used tyrosinase inhibitor, was discovered through the screening of 600 marine fungi [49].

There is no report of tyrosinase inhibitors from the genera *Cladosporium*, *Lulwoana*, and *Westerdykella*. It has been discovered that various *Penicillium* spp. can produce kojic acid, but there has been no report of any tyrosinase inhibitor from *P. citrinum*. On the other hand, several tyrosinase inhibitors have been reported from the genus *Trichoderma*, for example, homothallin II from *T. viride* [50] and 1-(1,2,5-trihydroxy-3-isocyanopent-3-enyl)-ethanol, 1-(3-chloro-1,2-dihydroxy-4-isocyano-4-cyclopenten-1-yl)ethanol, and 1-(1,2,3-trihydroxy-3-isocyano-4-cyclopenten-1-yl)ethanol from *T. harzianum* [51,52]. Some antioxidants have been reported to be able to inhibit tyrosinase by scavenging reactive quinone products [48]. Since none of the five fungal extracts exhibited remarkable radical-scavenging activity, it is obvious that their tyrosinase inhibitors use inhibiting mechanisms other than scavenging reactive quinone products.

Considering that all the activities were measured using crude extracts, the tyrosinase inhibitory activity of *C. sphaerospermum* KUC21388, *P. citrinum* KUC21390, and *Trichoderma harzianum* KUC21411 extracts were notable. In particular, the IC_50_ value of *C. sphaerospermum* KUC21388 extract was even comparable to that of the positive control, kojic acid. This is the first report of tyrosinase inhibition activity in the genus *Cladosporium*, which is generally known to produce melanin.

#### 2.2.3. Antifungal Activity

Antifungal experiments on *Asteromyces cruciatus* and *Lindra thalassiae* were conducted using the extracts of fungi isolated from intertidal mudflats and abandoned salterns. The two target fungi were selected considering that our fungal isolates were derived from the marine environment. *A*. *cruciatus* is a potentially harmful fungus to brown algae because it degrades alginate, a major constituent of brown algae. Alginate plays an important role in brown algae by forming the structure of the algal biomass and physically protecting algae from pathogens. *L. thalassiae* is a pathogenic fungus of sea plants and brown algae that causes raisin disease [53,54,55]. The results showed that the extracts of 17 fungal strains showed inhibitory effects against mycelial growth in *A. cruciatus* or *L. thalassiae* (Table 5).

All the extracts of the four *Penicillium* sp. 1 strains had potential antifungal activities, as they could inhibit the growth of *A. cruciatus* at a minimum concentration of 50 µg/mL and could inhibit the growth of *L. thalassiae* as well. Since they were identified as the same species from the same isolation source, the potential antifungal secondary metabolites are highly likely to be identical. There are a number of antifungal compounds reported from the genus *Penicillium*, for example, brefeldin A from *P. brefeldianum*; griseofulvin from *P. griseofulvum*; atpenins A4, A5, and B from *P. atramentosum* [56]; xanthocillin X and penicisteroid A from *P. chrysogenum* [56,57]; calbistrins from *P. restrictum* [58]; canadensolide from *P. canadense* [59]; macrocyclic polylactones from *P. verruculosum* [60]; patulin from *P. carneum* , citrinin from *P. melinii* (previously known as *P. damascenum*), palitantin and arthrographol from *P. implicatum* [61]; and compactin from *P. brevicompactum* [62]. There is no report of antifungal compounds or activity from the genus *Pseudeurotium*. In the case of the genus *Talaromyces*, some antifungal compounds have been reported, for example, talaroconvolutins from *T. convolutus* [63], talaron from *T. flavus* [64], 3-*O*-methylfunicone from *T. pinophilus*, wortmannin from *T. wortmannii*, macrophorin A from *T. purpurogenus*, and botryodiplodin from *T. stipitatus* [56]. In particular, *Penicillium* sp. 1 KUC21389 and *Pseudeurotium bakeri* KUC21422 exhibited significantly lower MIC values than the other strains. To the best of our knowledge, this is the first report of the antifungal activity of the genus *Pseudeurotium*. It is strongly speculated that these antifungal metabolites can affect other pathogenic fungi, so they could be developed as biocontrol agents.

#### 2.2.4. Quorum Sensing Inhibitory Activity

**Figure 6:**
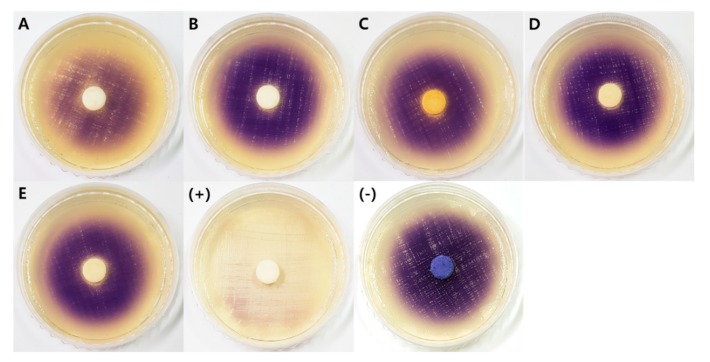
Quorum-sensing inhibitory activity of fungal extracts. (**A**) *Aspergillus urmiensis* KUC21392; (**B**) *Penicillium* sp. 2 KUC21400; (**C**) *Talaromyces stipitatus* KUC21402; (**D**) *Trichoderma afroharzianum* KUC21401; (**E**) *Trichoderma afroharzianum* KUC21404; (+) positive control (piericidin A); (−) negative control (DMSO).

Since only a few fungal quorum-sensing inhibitors (QSIs) have been found to date, it is worth investigating fungi from extreme environments such as intertidal mudflats and abandoned salterns. The two-step screening process showed that the extracts of *A. urmiensis* KUC21392 moderately inhibited the production of violacein by *C. violaceum* CV026 in the presence of N-(3-oxo-hexanoyl)-l-homoserine lactone (3-oxo-C6-HSL), an N-acyl homoserine lactone (AHL), which is one of the most widely studied quorum sensing (QS) molecules (Figure 6). Bacterial biofilms cause huge economic losses in many areas, such as the food industry, aquaculture, wastewater treatment, and the shipping industry [65,66]. QS is responsible for biofilm formation and the expression of virulence genes, and bacterial biofilms have been reported to be approximately 1,000 times more resistant to antibiotics than their planktonic counterparts [67]. Some fungi produce quorum-quenching compounds such as patulin and penicillic acid from *Penicillium* spp., which can suppress bacterial biofilm formation [68]. Other studies reported that the extracts of *Fusarium graminearum*, *Lasidiplodia* sp., *Phellinus igniarius*, and fruiting bodies of *Auricularia auricular*, *Tremella fuciformis*, and *Ganoderma lucidum* have QS inhibitory activity [69,70,71,72,73,74]. Fungal QSIs were reported in the genera *Penicillium* and *Trichoderma*: polyhydroxyanthraquinones, penicillic acid, and patulin from *P. restrictum*, *P. radicicola*, and *P. coprobium*, respectively [68,75]; and carot-4-en-9,10-diol from *T. virens* [76]. *Penicillium* spp. are a good source of QSIs, considering that 33 out of 100 *Penicillium* extracts were screened as potential QSI producers in a previous study. In addition, *Penicillium atramentosum* was reported to produce QSIs other than penicillic acid or patulin [77]. *A. urmiensis* KUC21392 was identified as a potential QSI producer and is valuable as a source of natural compounds that regulate bacterial biofilms and related diseases. This is the first report on the QS inhibitory activity of the genus *Aspergillus*.

Interestingly, four fungal extracts (*Penicillium* sp. 2 KUC21400, *T. stipitatus* KUC21402, *T. afroharzianum* KUC21401 and KUC21404) inhibited the growth of *C. violaceum*, resulting in a distinct hollow circle around the paper disc (Figure 5). Based on their antibacterial activity against *C. violaceum*, these four fungal strains were found to be producers of antimicrobial compounds that inhibit gram-negative bacteria. A number of antibacterial compounds have been discovered from the genus *Penicillium*, including rugulotrosin A and B, secalonic acid B and D, penicillixanthone A, citrinin, viridicatumtoxin B, penialidin B and C, penicyclones A–E [78,79,80,81,82,83]. *P. rubens* is the producer of penicillin, the first antibiotic that triggered the discovery of antibiotics [84]. Norlichexanthone, 3,1′-didehydro-3[2″(3′″,3′″-dimethyl-prop-2-enyl)-3″-indolylmethylene]-6-methyl pipera-zine-2,5-dione, gladiolic acid, and talaroderxines A and B were also produced by *Penicillium solitum*, *P. chrysogenum*, *Penicillium gladioli*, and *Penicillium derxii*, respectively [85,86,87,88]. The genus *Talaromyces*, which was formerly regarded as the teleomorphic state of *Penicillium*, has also been reported to produce various antibacterial compounds: fumitremorgin C, pseurotin A_1_ and A_2_, 3-dehydroxymethylbisdethio-3,10a-bis(methylthio)gliotoxin, bisdethiobis(methylthio)gliotoxin, didehydrobisdethiobis(methylthio)gliotoxin, 7-hydroxy-deoxytalaroflavone, deoxytalaroflavone, 7-epiaustdiol, 8-O-methylepiaustdiol, stemphyperylenol, skyrin, secalonic acid A, emodin, norlichexanthone, and talaromycesone A [89,90,91,92,93]; (−)luteoskyrin, cyclochlorotine, (+)rugulosin, rubroskyrin, lumiluteoskyrin, simotoxin, pibasterol, and skyrin produced from *T. islandicu*s and *Talaromyces rugulosus* [94]; wortmannin, flavomannins A-D, and talaromannins A and B from *T. wortmannii* [95,96]; (−)-8-hydroxy-3-(4-hydroxypentyl)-3,4-dihydroisocoumarin, pentalsamonin, 5-hydroxy-7-methoxy-2-methylbenzofuran-3-carboxylic acid and 1-(5-hydroxy-7-methoxybenzofuran-3-yl)ethan-1-one, bacillosporin A, and talaromycolides A–C from *Talaromyces verruculosus*, *T. purpureogenus*, *Talaromyces amestolkiae*, *Talaromyces bacillisporus*, and *T. pinophilus* respectively [45,97,98,99,100]; talaraculone B, (-)mitorubrin, and bacillisporin A and B from *Talaromyces aculeatus* [101,102]; and 2,2′,3,5′-tetrahydroxy-3′-methylbenzophenone, 1,4,7-trihydroxy-6-methylxanthone, and 1,4,5-trihydroxy-2-methylxanthone from *T. islandicus* [103]. In the case of *T. stipitatus*, talaromyone was reported as an antibacterial compound [104]. Therefore, the potential antibacterial compound produced by *T. stipitatus* KUC21402 is likely to be talaromyone. The genus *Trichoderma* is well known for producing various antibacterial compounds: 3-(3-oxocyclopent-1-enyl) propanoic acid, trichodermaol, alternariol 1′-hydroxy-9-methyl ether [105,106,107]; pseudokonin KL III and IV produced from *T. pseudokoningii* [108]; suzukacillin, viridian, lignoren, and gliotoxin from *T. viride* [109,110,111,112]; 6-n-pentyl-α-pyrone from *T. harzianum* and *T. longibrachiatum* [113]; shikimic acid from *T. ovalisporum* [114]; and coniothranthraquinone 1 and emodin from *T. aureoviride* [115]. Therefore, the potential antibacterial compounds of *T. afroharzianum* KUC21401 and KUC21404 are unlikely to be novel, but this is the first report on the antibacterial activity of this species.

## 3. Materials and Methods

### 3.1. Microorganisms and Diversity Analyses

#### 3.1.1. Sediment Sampling and Fungal Isolation

Intertidal sediments 10 cm deep from the surface were sampled into sterile polyethylene jars using a sterile knife. The sediment samples were sieved using 2-mm sieves and stored at −80 °C for further use. To isolate fungi from the sediment samples, 1 gram of wet sediment was mixed with 10 mL of sterilized D.W. and vortexed. Preliminary tests with a variety of different intertidal sediment samples showed that the optimal concentration for isolating fungi from intertidal sediments was 5–10 times higher than those typically used when using forest sediments. The reason may be that the amount of organic matter in the sediment is relatively small, so that the amount of microbial biomass is small. The sediment suspension was serially diluted to a ratio of 1 g : 1000 mL, and 3 mL of the diluted suspension was added to 47 mL of the culture medium composed of 2% malt extract, 3.59% artificial sea salt (Instant Ocean, Aquarium Systems, Mentor, OH, USA), and 0.01% streptomycin sulfate (Sigma-Aldrich, St. Louis, MO, USA) for inhibiting the growth of bacteria. After that, 150 µL of the inoculated medium was distributed into each well of four sterilized 96-well plates. The plates were incubated at room temperature for 2–4 weeks until the fungal mycelia were visible to the naked eye. Each fungal colony was transferred to an individual 2% malt extract agar plate with 0.01% streptomycin sulfate. The plates were incubated at room temperature for a week and subcultured to isolate single strains. The isolated fungal strains were stored in 10% glycerol solution at 4 °C for further use and deposited in the Korea University Culture (KUC) collection.

#### 3.1.2. DNA Extraction, PCR, and Identification

Genomic DNA was extracted from fungal culture using the *AccuPrep*^®^ Genomic DNA Extraction Kit (Bioneer, Seoul, Republic of Korea), of which lysis buffer was replaced with fungal lysis buffer (12 mL of 0.5 M EDTA pH 8.0, 40 mL of 1 M Tris-HCL pH 8.0, 1 g of SDS, 0.8766 g of NaCl, and D.W. to a final volume of 100 mL). PCR amplification of ITS regions were carried out with ITS1F-LR3 primer [ITS1F (5′-CTT GGT CAT TTA GAG GAA GTA A-3′) and LR3 (5′- CCG TGT TTC AAG ACG GG-3′) [116,117] using the *AccuPower^®^* PCR Premix (Bioneer, Seoul, Republic of Korea). *benA* for *Talaromyces* species was amplified using the Bt2a-Bt2b primer [Bt2a (5′-GGT AAC CAA ATC GGT GCT GCT TTC-3′) and Bt2b (5′-ACC CTC AGT GTA GTG ACC CTT GGC-3′)] [118]. EF1-α for *Trichoderma* species was amplified using EF1-EF2 primer [EF1 (5’-ATG GGT AAG GAR GAC AAG AC-3’) and EF2 (5’-GGA RGT ACC AGT SAT CAT GTT-3’)] [119]. ITS regions were amplified under the conditions: 95 °C for 4 min, followed by 35 cycles of 95 °C for 30 s, 54 °C or 55 °C for 30 s, and 72 °C for 30 s. An elongation step of 72 °C for 5 min was performed at the end. For *benA*, the conditions were as follows: 95 °C for 5 min, followed by 38 cycles of 95 °C for 35 s, 55 °C or 56 °C for 50 s, and 72 °C for 2 min; an elongation step was performed at 72 °C for 7 min. For EF1-α, the conditions were as follows: 94 °C for 2 min, followed by 34 cycles of 93 °C for 30 s, 56 °C for 30 s, and 72 °C for 1 minute; and an elongation step was performed at 72 °C for 10 min. Through electrophoresis with 1% agarose gel, PCR products were conformed. PCR products were purified with the *AccuPrep*^®^ PCR Purification Kit (Bioneer, Seoul, Republic of Korea) according to the user guide. DNA sequencing was performed using an 3730xl DNA Analyzer (Life technology, Carlsbad, CA, USA) with indicated PCR primers by Macrogen (Seoul, Republic of Korea). All the sequences were submitted to the GenBank and are presented in Table 1.

#### 3.1.3. Phylogenetic Analysis

All the obtained sequences were assembled, proofread and edited using MEGA v. 7.0 [120]. Edited sequences were aligned using MAFFT 7.130 [121] and ambiguously aligned positions manually modified using MacClade 4.08 [122]. Through MrModeltest 2.3 using Akaike information criterion criteria, the best-fit models were calculated for the Bayesian phylogenetic analysis [123]. The Bayesian phylogenetic analyses were performed for 10 million generations using MrBayes 3.2.1 [124]. Phylogenetic trees were sampled every 100^th^ generation. After sampling, the last 75% of the trees were selected. The constructed phylogenetic tree followed the 50% majority-rule and the tree reliability was presented by posterior probability.

#### 3.1.4. DNA library Preparation and Amplicon Sequencing

Total DNA was extracted from 0.3 g of frozen sediment using a PowerSoil DNA isolation kit (MoBio, Carlsbad, CA, USA) following the manufacturer’s protocol. DNA libraries were constructed using the Illumina MiSeq platform with the fungal ITS rDNA gene. An approximate 300–350 bp region of the ITS2 region was amplified with forward primer fITS7 [125] and reverse primer ITS4 [116]. Primer fITS7 contained a unique 12-nt barcode at the 5′ end for MiSeq sequencing detection. Paired-end read sequences were generated by high-throughput sequencing technology.

#### 3.1.5. Bioinformatics Analyses

The sequences obtained in this study were processed using QIIME v1.9.1 [126]. The primer, key, and barcode sequences were trimmed from both ends. Sequences composed of homopolymers (n > 6) and chimeras were removed, and the remaining sequences were analyzed. The sequences were then clustered as operational taxonomic units (OTUs) based on a ≥ 97% similarity threshold and the average linkage method using Vsearch [127]. The representative sequence that was most abundant from each OTU was taxonomically assigned using the UNITE [128] reference database.

Nonmetric multidimensional scaling (NMDS) analysis applying the Bray–Curtis similarity index was performed to plot the similarity of the fungal communities in a way such that distances could be represented in two dimensions, using the vegan package in R statistical software, version 3.5.3. Alpha diversity indices were calculated using QIIME, and differences among the sediments from the abandoned salterns and mudflats were compared. After taxonomic profiling, we also investigated which fungal taxa differed in relative abundance among the intertidal environments. The relative abundances of the fungal communities were compared using linear discriminant analysis effect size (LefSe) analysis [129] with a LDA score threshold of 2.0 at the phylum, class, order, and family levels. Indicator species analysis was performed using the labdsv package in R to determine the key fungal taxa that best represent the Yellow Sea intertidal environments. Indicator species values are based on how specific and widespread the taxa are within a particular group and are independent of the relative abundance of other fungi [130]. RandomForest analysis was performed to identify key predictors of the environments among the fungal taxa. The accuracy importance measure was calculated for each tree and averaged over the forest (10,000 trees). This analysis was performed using the randomForest package in R. To determine which fungal taxa differed among the environments, the multivariate statistical package mvabund [131] was used in R; this approach is reported to have much greater power than distance-based approaches. In the generalized linear model, the relative abundance of each fungal taxon was modeled on the negative binomial distribution.

### 3.2. Preparation of Fungal Extracts

All the fungal isolates were precultured on 90-mm Petri dishes (10090, SPL Life Sciences Co., Pocheon, Republic of Korea) containing 20 mL of potato dextrose agar (PDA) at 25 °C for a week. After that, three agar plugs with actively-growing mycelia were inoculated to 150-mm Petri dishes (10150, SPL Life Sciences Co., Pocheon, Republic of Korea) containing 50 mL of PDA and incubated at 25 °C for 7 days in the dark. The cultures were extracted with 200 mL of MeOH and filtered with Whatman No.1 filter paper. The filtrates were dried at 35 °C using a rotary evaporator, and the dried extracts were dissolved in 15 mL of D.W. and 15 mL of EtOAc. After 6 hours, the EtOAc layers were transferred to 20-mL scintillation vials and dried using the rotary evaporator, and the dried extracts were stored at 4 °C until use. All the extracts were prepared in triplicate.

### 3.3. Biological Assays

The biological assays were performed with methods that were the same as or slightly modified from the methods used in our previous study [132].

#### 3.3.1. Antioxidant Assay

##### ABTS Radical-Scavenging Assay

The 7-mM ABTS (Sigma-Aldrich, St. Louis, MO, USA) solution in phosphate-buffered saline (PBS, pH 7.4) was mixed with potassium persulfate solution that dissolved in PBS to 2.45 mM. The mixture was stored in the dark at room temperature for 24 h to form the ABTS^•+^. The solution was diluted with PBS to an absorbance of 0.70 (±0.02) at a wavelength of 734 nm. Then, 198 µL of ABTS radical solution was mixed with 2 µL of the fungal extract (10 mg/mL DMSO) in a 96-well plate. The absorbance was measured at 734 nm after 6 min using a spectrophotometer (Sunrise^TM^, Tecan Group Ltd., Port Melbourne, VIC, Australia). Ascorbic acid (Sigma Aldrich, St. Louis, MO, USA) was used as a positive control.

##### DPPH Radical-Scavenging Assay

The 200 µL of 150 µM DPPH (Sigma-Aldrich, St. Louis, MO, USA) solution in 80% methanol was mixed with 22 µL of the fungal extract (10 mg/mL DMSO) in a 96-well plate. The plate was stored in the dark at room temperature, and the absorbance was measured at 540 nm after 30 min using the spectrophotometer. Ascorbic acid was used as a positive control.

#### 3.3.2. Tyrosinase Inhibition Assay

Tyrosinase inhibitory activity was measured based on the method of Lai et al. (2009) [133]. Briefly, 70 μL of 0.1 M potassium phosphate buffer (pH 6.8), 40 μL of fungal extract (2.5 mg/mL 50% DMSO), and 30 μL of tyrosinase from mushroom (0.02 mg/mL buffer; Sigma Aldrich, St. Louis, MO, USA) were added into each well of the 96-well plate. The plates were floated on water in a waterbath at 30 °C for 5 minutes, and 100 μL of 2.5 mM l-DOPA (Sigma Aldrich, St. Louis, MO, USA) was added. After 30 min, the plates were placed in ice to terminate the reaction, and the absorbance was measured at 492 nm. Kojic acid (Sigma Aldrich, St. Louis, MO, USA) was used as a positive control.

#### 3.3.3. Antifungal Assay

*Asteromyces cruciatus* SFC20161110-M19 was obtained from Marine Fungal Resource Bank (MFRB) at Seoul National University as a marine bioresource bank of Korea by the Ministry of Oceans and Fisheries, and *Lindra thalassiae* NBRC106646 was purchased from Biological Resource Center under National Institute of Technology and Evaluation. Antifungal activity was determined in a 96-well plate using the microtiter broth dilution method [134]. Twenty-five microliters of spore suspensions (4 × 10^5^ conidia/mL) of the target fungi were added to each well containing 25 µL of 4X potato dextrose broth and 49 µL of D.W. The fungal extracts were added to a final concentration of 100 µg/mL. The inoculated plates were incubated at 25 °C for 3 days. The extracts with lower concentrations (50, 25, 12.5, 6.5 µg/mL) were tested to determine MIC, the minimum concentration of an antimicrobial agent that causes microbial death.

#### 3.3.4. Quorum Sensing Inhibition Assay

QSI screening was performed based on the inhibition of violacein production by *Chromobacterium violaceum* CV026 strains under culture conditions supplemented with an exogenous QS molecule, 3-oxo-C6-HSL [135]. The *C. violaceum* CV026 cultured overnight was diluted with LB medium (5 g of yeast extract, 10 g of tryptone, and 5 g of NaCl in a liter of D.W.) to an OD_600 nm_ of 0.1, and 97 µL of the diluted culture was added to each well of a 96-well plate. One microliter of 3-oxo-C6-HSL (final concentration of 10 µM; Sigma–Aldrich, St. Louis, MO, USA) and 2 microliters of fungal extract (10 mg/mL DMSO) were added to each well. As a negative and positive control, two microliters of DMSO and two microliters of 100 nM (*Z*)-4-bromo-5-(bromomethylene)-2(5*H*)-furanone (Furanone C-30; Sigma-Aldrich, St. Louis, MO, USA) were used, respectively. After 16 h at 28 °C, 100 µL of DMSO was added to each well and vigorously shaken for an hour at room temperature to determine the production of violacein.

The selected extracts were further examined using a paper disc method. Fifteen microliters of the diluted culture of *C. violaceum* CV026 was spread on an LB agar plate, and a paper disc (diameter 8 mm; Advantec, Tokyo, Japan) impregnated with 40 μL of extract and 1 μL of 1 mM 3-oxo-C6-HSL was placed in the center of the plate. DMSO was used as a negative control, and piericidin A isolated from *Streptomyces xanthocidicus* KPP01532 was used as a positive control [135]. The plates were incubated overnight at 28 °C.

### 3.4. Statistical Analyses

The half maximal inhibitory concentration (IC_50_) values were calculated by nonlinear regression analysis using SigmaPlot 12.0 (Systat Software Inc., San Jose, CA, USA).

## 4. Conclusions

The results of the fungal community analysis showed that only the abandoned saltern in Yongyudo was significantly different from the other abandoned salterns or mudflats. The fungal communities in the abandoned salterns in Yubudo and Gopado had been restored to a similar status as those in the mudflats, but that was not the case of the saltern in Yongyudo, which was abandoned less than a year ago. The results also implied that it takes more than 35 years to restore abandoned salterns to mudflats via natural restoration. The α-diversity of the fungal community in the NS was significantly lower than that of the RSs and mudflats. The diversity indices were high in general, which implies that these unique environments are valuable for bioprospecting.

Through various statistical analyses, the lineages of Gigasporales sp. and *Umbelopsis* sp. were selected as indicator taxa of mudflats in the Yellow Sea in South Korea. We demonstrate that the ecological recovery of the abandoned salterns can be assessed by using the two indicator taxa to indicate not only the recovery state but also the recovery direction, i.e., whether the recovery process is moving in the right direction. In this study, several analytical methods were applied to investigate indicator taxa. However, the results were not identical, even though we only used methods that use the absolute abundance data rather than processed data such as ecological distances that could lead to statistical misinterpretation [27]. Therefore, indicator taxa must be selected by carefully comparing the results of various types of analysis. We also proved that the fungal community is highly suitable for use in ecosystem assessment, as it has a sufficient level of identification with good resolution. The limitation was that the number of samples, especially for the NS and RS environments, was insufficient. A larger sample size is recommended for more accurate statistical analysis with high fidelity.

To evaluate the fungal diversity and compare it with the results from the metagenome analysis, the culturable fungi were isolated from the sediment samples. A total of 53 fungal strains was isolated, and the number of fungal isolates differed by sampling location rather than by environment type. Based on the phylogenetic analysis, *Talaromyces* was the most diverse genus, consisting of seven different species. The genera *Talaromyces* and *Trichoderma* were mostly isolated from the mudflat sediments, and most *Penicillium* spp. were found in the NS. The diversity and the number of isolates in the NS were higher than those in the other salterns, which is inconsistent with the results of metagenomics, and every isolate except *P. chrysosporium* belongs to the Ascomycota. This result showed that metagenomics must be applied when examining the microbial diversity within an actual environment, as has been suggested over the past decades [29]. Interestingly, a total of 18 fungal isolates was identified as candidate novel species.

To evaluate the potential value of the intertidal environments for bioprospecting, a variety of biological activities of the fungal extracts were investigated. Regardless of the actual diversity, the isolates from both intertidal environments exhibited a variety of biological activities. Over half of the culturable fungal isolates (29 strains) exhibited biological activities, and *Penicillium* spp. exhibited the most varied activities. The antioxidant compound of *Phoma* sp. KUC21426, the tyrosinase inhibitors of *C. sphaerospermum* KUC21388, the antifungal compounds of *Penicillium* sp. 1 KUC21389 and *P. bakeri* KUC21422, and the quorum-sensing inhibitor of *A. urmiensis* KUC21392 will be separated and identified in the near future. Since bioactive compounds with these activities have never been reported from these species, the likelihood of discovering novel compounds is high.

In this study, we presented the fungal community and diversity of the abandoned solar salterns and the intertidal mudflats on three different islands located in the Yellow Sea of South Korea, and we identified indicator taxa for the assessment of the restoration of abandoned salterns to mudflats. Additionally, we provided reliable DNA information for the 53 fungal isolates from the intertidal sediments and their exploitable biological activities. We demonstrated that these unique environments are highly valuable in bioprospecting not only for novel microorganisms but also for novel bioactive compounds. Restoring the reduced biodiversity of abandoned salterns is important in terms of environmental protection and conservation. However, many novel fungal species were found from the abandoned salterns, and interestingly, all fungal strains selected for high bioactivity were isolated from NS or RS. Therefore, active measures to recover abandoned salterns to mudflats should be carefully determined and planned, taking into account their value as a source of biological resources.

## Figures and Tables

**Table 1:** The α-diversity indices of the fungal community in abandoned salterns and intertidal mudflats.

Sample	Shannon-Wiener Index (H’)	Gini-Simpson Index (D^S^, 1-λ)
**Gopado**		
Abandoned saltern **(RS)**	4.177	0.928
Mudflat	3.607	0.885
**Yubudo**		
Abandoned saltern **(RS)**	4.433	0.954
Mudflat	3.901	0.806
**Yongyudo**		
Abandoned saltern **(NS)**	1.471*	0.477*
Mudflat	3.497	0.859

Mean values are presented. NS, non-recovered saltern; RS, recovered saltern. * statistical significance of *p* < 0.05.

**Table 2:** Indicator taxa results from the indicator species analysis (IndVal score > 0.9, *p* < 0.05, and Frequency ≥ 5).

Taxa	Group	IndVal Score	*p* Value	Frequency
**Ascomycota**				
*Stemphylium*	NS	1.0000	0.005	8
Lecanoromycetes	NS	0.9012	0.007	17
Pertusariales	NS	0.9266	0.005	16
**Basidiomycota**				
Chionosphaeraceae	RS	0.9137	0.005	15
*Ballistosporomyces sasicola*	RS	0.9137	0.006	13
Tremellales	NS	0.9238	0.005	12
Tremellaceae	NS	0.9362	0.004	12
*Tremella*	NS	0.9359	0.005	9
**Chytridiomycota**				
*Zygorhizidium planktonicum*	RS	0.9322	0.003	8
**Entorrhizomycota**	NS	0.9995	0.004	16
Entorrhizomycetes	NS	0.9995	0.003	16
**Mucoromycota**				
Umbelopsidomycetes	Mudflat	0.9553	0.001	5
*Umbelopsis* sp.	Mudflat	0.9632	0.001	5

NS, non-recovered saltern; RS, recovered saltern.

**Table 3:** Indicator taxon results from the mvabund analysis (*p* < 0.05).

Taxa	Dev.	*p* Value
**Ascomycota**		
*Leptosporella*	26.15	0.035
**Basidiomycota**		
Agaricostilbomycetes	23.23	0.011
**Chytridiomycota**	21.43	0.011
Rhizophydiales	33.15	0.008
Rhizophydiaceae	31.84	0.013
Chytridiomycetes	23.13	0.013
**Entorrhizomycota**	34.62	0.004
Entorrhizomycetes	34.62	0.003
Entorrhizaceae	30.59	0.015
*Entorrhiza cypericola*	27.29	0.001
**Glomeromycota**		
Gigasporales	36.90	0.005
**Mucoromycota**		
Umbelopsidomycetes	22.20	0.018
Umbelopsidaceae	23.36	0.047
**Rozellomycota**	16.23	0.033

**Table 4:** General information on the 53 fungal isolates from sediments of abandoned salterns and intertidal mudflats.

Fungal Name	KUC ID	Isolation Source		GenBank Accession Number		
		Location	Environmental Type	ITS	*benA*	EF1-α
*Acremonium* sp.	21384	Yongyudo	Abandoned saltern (NS)	MN518379	N.D.	N.D.
*Aspergillus urmiensis*	21379			MN518380	N.D.	N.D.
*A. urmiensis*	21392			MN518381	N.D.	N.D.
*A. urmiensis*	21396			MN518382	N.D.	N.D.
*Cladosporium sphaerospermum*	21388			MN518383	N.D.	N.D.
*Emericellopsis microspora*	21381			MN518384	N.D.	N.D.
*Gibellulopsis nigrescens*	21385			MN518385	N.D.	N.D.
*Humicola alopallonella*	21393			MN518386	N.D.	N.D.
*Lulwoana* sp.	21398			MN518387	N.D.	N.D.
Nectriaceae sp.	21383			MN518388	N.D.	N.D.
*Neocamarosporium betae*	21428			MN518389	N.D.	N.D.
*Penicillium chrysogenum*	21395			MN518390	N.D.	N.D.
*Penicillium citrinum*	21390			MN518391	N.D.	N.D.
*Penicillium sumatrense*	21382			MN518392	N.D.	N.D.
*Penicillium* sp. 1	21380			MN518393	N.D.	N.D.
*Penicillium* sp. 1	21386			MN518394	N.D.	N.D.
*Penicillium* sp. 1	21387			MN518395	N.D.	N.D.
*Penicillium* sp. 1	21389			MN518396	N.D.	N.D.
*Penicillium* sp. 1	21399			MN518397	N.D.	N.D.
*Phanerochaete chrysosporium*	10791			MN518398	N.D.	N.D.
*Simplicillium aogashimaense*	21397			MN518399	N.D.	N.D.
Sporormiaceae sp.	21391			MN518400	N.D.	N.D.
*Trichoderma harzianum*	21394			MN518401	N.D.	MN580170
*Acremonium persicinum*	21416		Mudflat	MN518402	N.D.	N.D.
*Amorphotheca resinae*	21414			MN518403	N.D.	N.D.
*Aspergillus floccosus*	21405			MN518404	N.D.	N.D.
Helotiaceae sp.	21409			MN518405	N.D.	N.D.
*Penicillium* sp. 2	21400			MN518406	N.D.	N.D.
*Pseudogymnoascus pannorum*	21417			MN518407	N.D.	N.D.
*Sporothrix mexicana*	21410			MN518408	N.D.	N.D.
*Talaromyces liani*	21412			MN518409	MN531288	N.D.
*Talaromyces stipitatus*	21402			MN518410	MN531294	N.D.
*Talaromyces* sp. 2	21413			MN518411	MN531292	N.D.
*Talaromyces* sp. 3	21415			MN518412	MN531289	N.D.
*Talaromyces* sp. 4	21408			MN518413	MN531293	N.D.
*Trichoderma koningiopsis*	21403			MN518414	N.D.	MN580171
*Trichoderma afroharzianum*	21401			MN518415	N.D.	MN580166
*T. afroharzianum*	21404			MN518416	N.D.	MN580167
*T. afroharzianum*	20406			MN518417	N.D.	MN580168
*Trichoderma harzianum*	21411			MN518418	N.D.	MN580169
*Westerdykella capitulum*	21407			MN518419	N.D.	N.D.
*Cladosporium* sp.	21418	Gopado	Abandoned saltern (RS)	MN518420	N.D.	N.D.
*Leptosphaeria* sp.	21419			MN518421	N.D.	N.D.
Hypocreales sp.	21420		Mudflat	MN518422	N.D.	N.D.
*Talaromyces* sp. 3	21421			MN518423	MN531290	N.D.
Aspergillaceae sp.	21424	Yubudo	Abandoned saltern (RS)	MN518424	N.D.	N.D.
*Aspergillus japonicus*	21425			MN518425	N.D.	N.D.
*Aspergillus tritici*	21427			MN518426	N.D.	N.D.
*Paracamarosporium* sp.	21423			MN518427	N.D.	N.D.
*Phoma* sp.	21426			MN518428	N.D.	N.D.
*Pseudeurotium bakeri*	21422			MN518429	N.D.	N.D.
*Talaromyces* sp. 1	21276			MN518430	MN531291	N.D.
*Monascus* sp.	21277		Mudflat	MN518431	N.D.	N.D.

KUC ID, Korea University Culture collection ID; ITS, internal transcribed spacer; *benA*, β-tubulin; EF1-α, translation elongation factor 1-α; NS, non-recovered saltern; RS, recovered saltern; N.D., no data.

**Table 5:** Fungal isolates from sediments of abandoned salterns and intertidal mudflats exhibiting radical-scavenging, tyrosinase inhibitory, and antifungal activity.

FUNGAL NAME	KUC ID	Antioxidant Activity		Tyrosinase Inhibitory Activity	Antifungal Activity	
		ABTS	DPPH		*Asteromyces cruciatus*	*Lindra thalassiae*
		(IC_50_, μg/mL)			(MIC, μg/mL)	
*Acremonium persicinum*	21416	> 50	> 1000	N.D.	N.D.	> 100
*Aspergillus floccosus*	21405	21.01	84.65	N.D.	N.D.	100
*Aspergillus japonicus*	21425	12.29	69.06	N.D.	N.D.	> 100
*Aspergillus urmiensis*	21396	22.75	105.67	N.D.	N.D.	N.D.
*Cladosporium sphaerospermum*	21388	> 50	> 500	66.57	N.D.	N.D.
*Lulwoana* sp.	21398	> 50	> 1000	> 417	N.D.	N.D.
*Paracamarosporium* sp.	21423	> 50	> 500	N.D.	N.D.	> 100
*Penicillium chrysogenum*	21395	56.75	214.14	N.D.	N.D.	N.D.
*Penicillium citrinum*	21390	> 50	> 1000	96.06	N.D.	N.D.
*Penicillium* sp. 1	21380	> 50	> 500	N.D.	50.00	> 100
*Penicillium* sp. 1	21386	10.24	102.53	N.D.	12.50	> 100
*Penicillium* sp. 1	21387	16.34	91.48	N.D.	50.00	100
*Penicillium* sp. 1	21389	11.88	101.30	N.D.	< 6.25	25
*Penicillium sumatrense*	21382	> 50	> 1000	N.D.	N.D.	> 100
*Phoma* sp.	21426	6.31	40.41	N.D.	N.D.	N.D.
*Pseudeurotium bakeri*	21422	> 50	> 1000	N.D.	< 6.25	100
*Talaromyces liani*	21412	27.58	208.09	N.D.	> 100	> 100
*Talaromyces* sp. 1	21276	> 50	> 1000	N.D.	N.D.	> 100
*Talaromyces* sp. 3	21415	> 50	> 1000	N.D.	> 100	50
*Talaromyces* sp. 3	21421	> 50	> 1000	N.D.	> 100	100
*Trichoderma harzianum*	21394	> 50	> 500	N.D.	N.D.	> 100
*T*. *harzianum*	21411	> 50	> 500	121.00	N.D.	> 100
*Westerdykella capitulum*	21407	> 50	N.D.	> 417	N.D.	N.D.
Ascorbic acid *		13.70	6.80			
Kojic acid *				49.32		

KUC ID, Korea University Culture collection ID; ABTS, 2.2’-azino-bis-3-ethylbenzothiazoline-6-sulfonic acid; DPPH, 2,2-diphenyl-1-picrylhydrazyl; IC_50_, half maximal inhibitory concentration; MIC, minimum inhibitory concentration; N.D., not detected. * positive controls.

## Supplementary Materials

The following are available online at https://www.mdpi.com/1660-3397/17/11/601/s1, Figure S1: Linear discriminant analysis (LDA) scores of the fungal taxa of which score > 2. NS and RS mean non-recovered saltern and recovered saltern, respectively, Figure S2: Relative abundance of Ascomycota in the three different intertidal environments. NS and RS mean non-recovered saltern and recovered saltern, respectively, Figure S3: The Bayesian analysis tree based on EF1-α for *Trichoderma* complex. Bayesian posterior probabilities (PP) at the nodes are presented if > 75. All of reference strains are type and ex-type strains. The fungi isolated from this study are in bold. The scale bar means the number of nucleotide substitutions per position, Figure S4: The Bayesian analysis tree based on combined of ITS and *benA* for *Talaromyces* complex. Bayesian posterior probabilities (PP) at the nodes are presented if > 75. Type strains are indicated as *. The fungi isolated from this study are in bold. The scale bar means the number of nucleotide substitutions per position, Table S1: The α-diversities of culturable fungal isolates from abandoned salterns and intertidal mudflats.
