# Supplementary material for: Fungal Diversity in Intertidal Mudflats and Abandoned Solar Salterns as a Source for Biological Resources
Source: Mar Drugs. 2019 Oct 23;17(11):601. doi: 10.3390/md17110601 (PMC6891761; doi:10.3390/md17110601)
Supplement: Supplementary file 1 [file marinedrugs-17-00601-s001.pdf]

Table S1. The α-diversities of culturable fungal isolates from abandoned salterns and intertidal mudflats.

**Sample**	**Shannon-Wiener index (H')**	**Gini-Simpson index (D^S, 1-λ)**
**Gopado**		
Abandoned saltern **(Recovered)**	0.693	0.500
Mudflat	0.693	0.500
**Yubudo**		
Abandoned saltern **(Recovered)**	1.946	0.857
Mudflat	0.000	0.000
**Yongyudo**		
Abandoned saltern **(Non-recovered)**	2.642	0.907
Mudflat	2.707	0.926

Mean values were presented. * statistical significance of $p < 0.05$

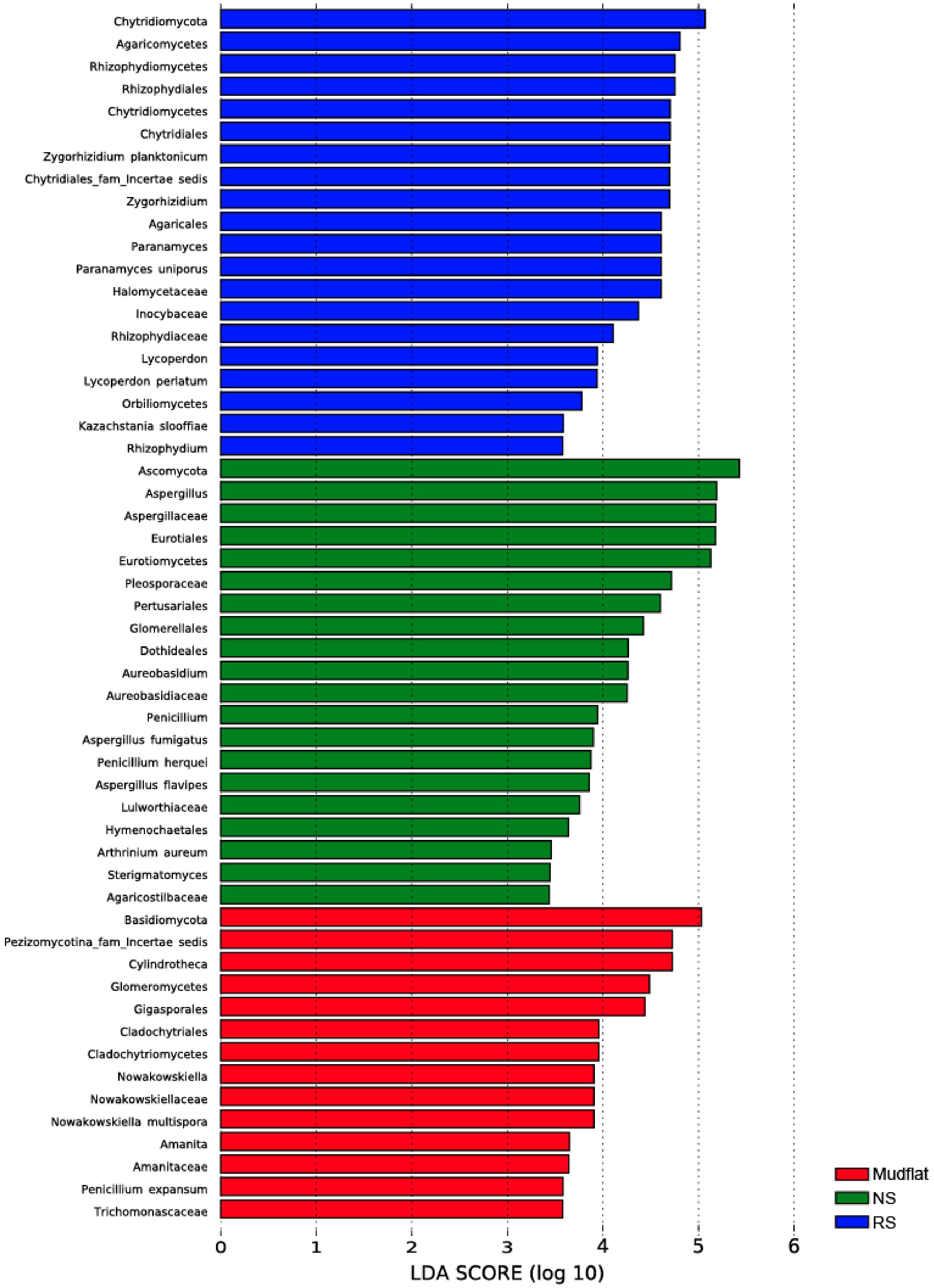

Figure S1. Linear discriminant analysis (LDA) scores of the fungal taxa of which score > 2. NS and RS mean non-recovered saltern and recovered saltern, respectively.

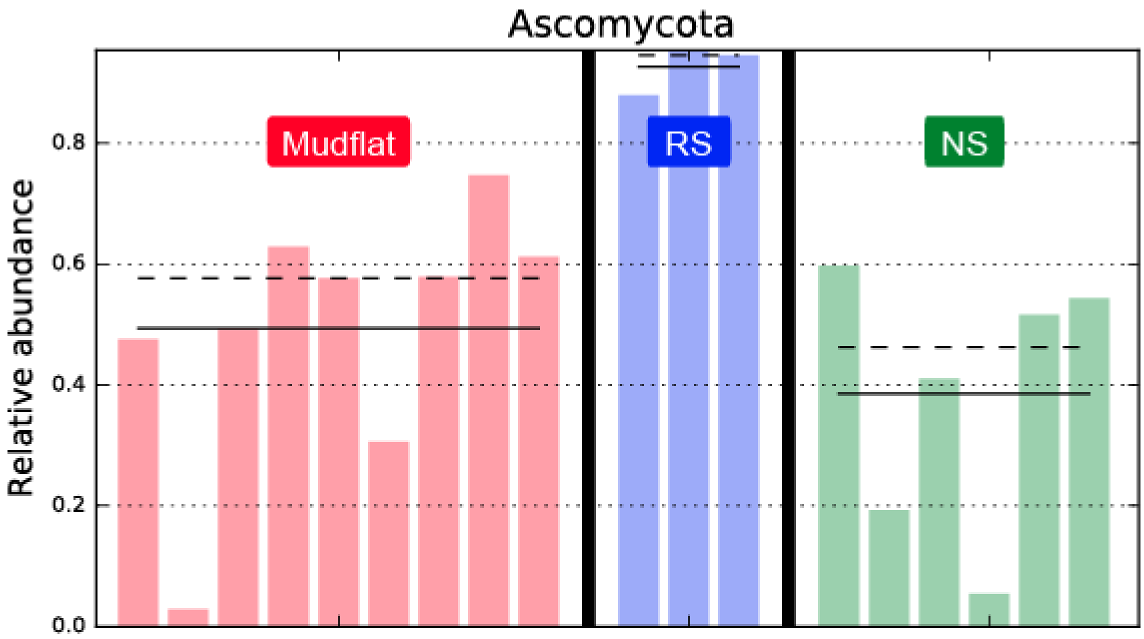

Figure S2. Relative abundance of Ascomycota in the three different intertidal environments. NS and RS mean non-recovered saltern and recovered saltern, respectively.

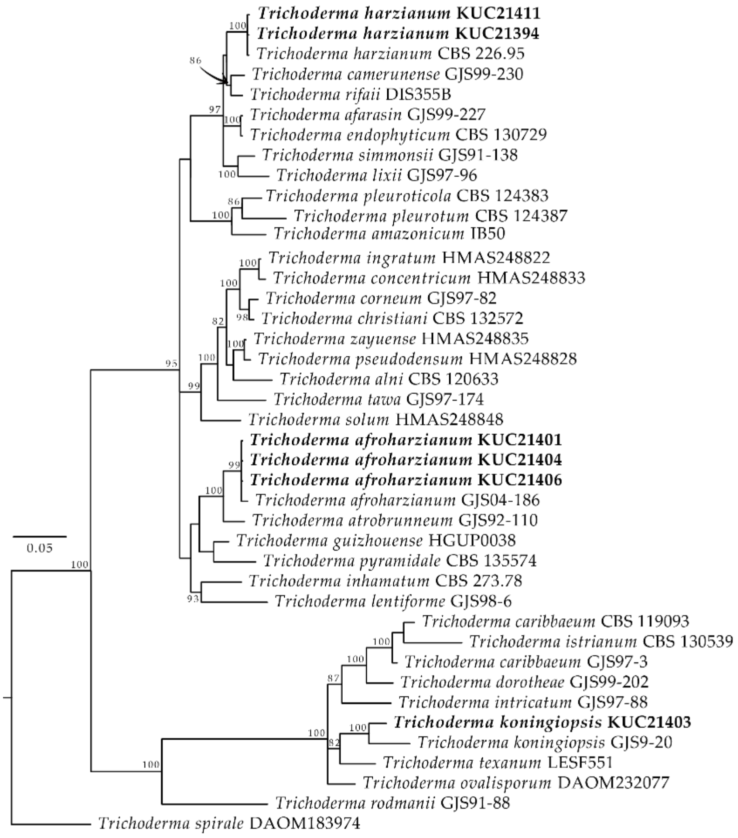

Figure S3. The Bayesian analysis tree based on EF1-α for *Trichoderma* complex. Bayesian posterior probabilities (PP) at the nodes are presented if > 75. All of reference strains are type and ex-type strains. The fungi isolated from this study are in bold. The scale bar means the number of nucleotide substitutions per position.

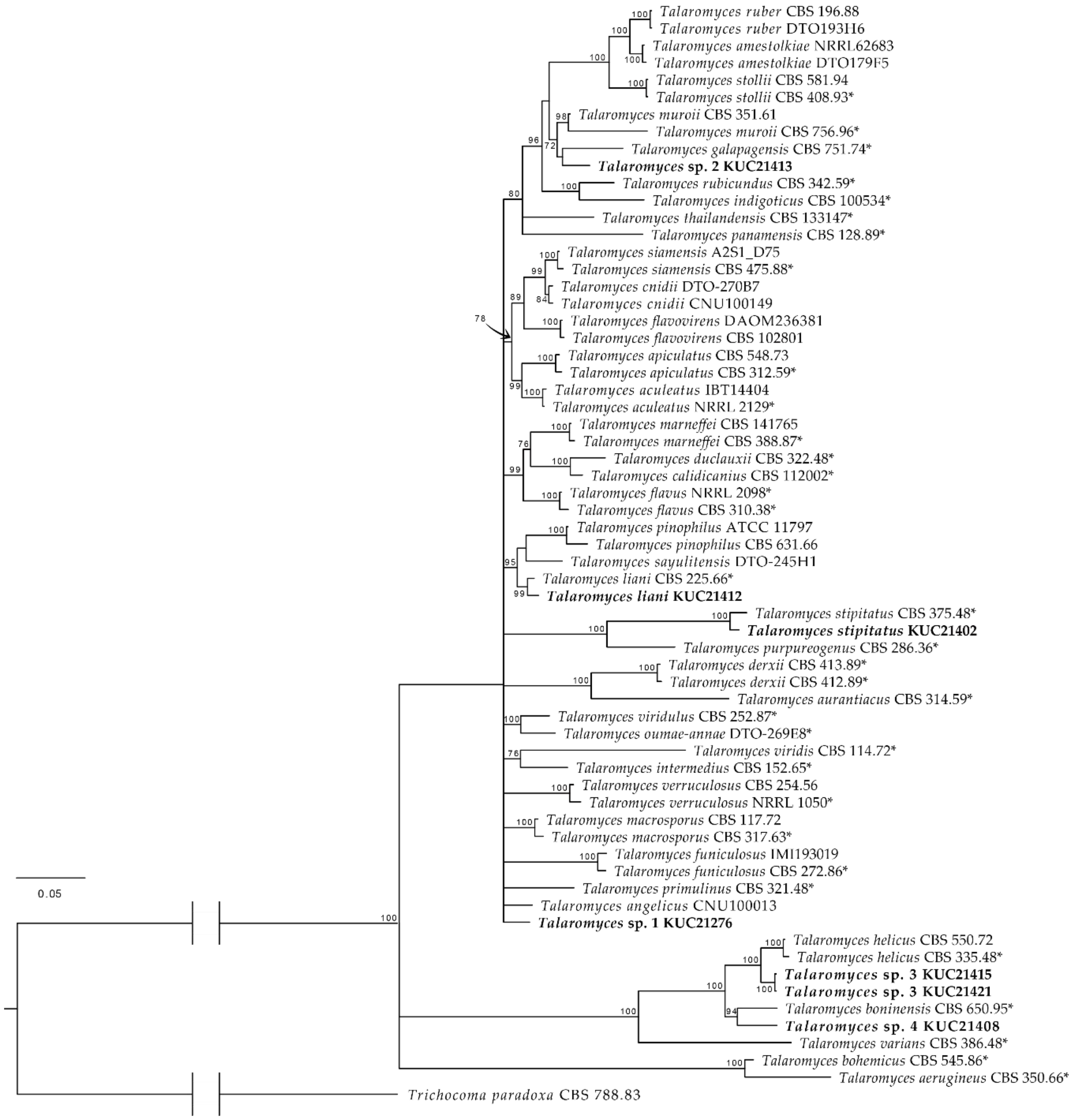

Figure S4. The Bayesian analysis tree based on combined of ITS and *benA* for *Talaromyces* complex. Bayesian posterior probabilities (PP) at the nodes are presented if > 75. Type strains are indicated as *. The fungi isolated from this study are in bold. The scale bar means the number of nucleotide substitutions per position.
